# Preliminary study on the inhibitory effect of seaweed *Gracilaria verrucosa* extract on biofilm formation of *Candida albican*s cultured from the saliva of a smoker

**DOI:** 10.12688/f1000research.14879.3

**Published:** 2018-09-18

**Authors:** Zaki Mubarak, Adintya Humaira, Basri A. Gani, Zainal A. Muchlisin

**Affiliations:** 1 Faculty of Dentistry, Syiah Kuala University, Banda Aceh, 23111, Indonesia; 2 Faculty of Marine and Fisheries, Syiah Kuala University, Banda Aceh, 23111, Indonesia

**Keywords:** Candida albicans, oral candidiasis, seaweed Gracilaria verrucosa

## Abstract

**Background:** *Candida albicans *is an opportunistic fungus that might infect the oral cavity. Increased colony numbers of *C. albicans *in the mouth can be caused by multiple factors, such as smoking, weakened immune system, antibiotics use and immune-compromised condition. Smoking can increase expression of virulence factors of *C. albicans* and make it stronger. One virulence factor of *C. albicans *is biofilm formation. The ability of creating biofilm makes *C. albicans *more tolerant to commercial antifungal agents. The objective of this preliminary study was to examine the ability of the seaweed *G.* *verrucosa *extracts to inhibit the formation of biofilm by *C. albicans* isolated from the saliva of a smoker.

**Methods:** The extract of *G. verrucosa* was prepared by maceration using 96% methanol and subjected for phytochemical analysis. *C. albicans *was isolated from the saliva of a smoker who voluntarily participated in the study after providing informed consent. In triplicate, the fungus was cultured in the growth medium containing increased concentrations of *G. verrucosa *(6.25, 12.5, 25, 50, 75 and 100% ).The same reaction using fluconazole 0.31 µg/ml *C. albicans* was prepared as positive control. Biofilm formation was accessed based on optical density of cell mixtures using an ELISA reader. The data obtained were subjected to Kruskal-Wallis test at a significance limit of 0.05.

**Results:** Methanol extract of seaweed *G. verrucosa *contained three bio-active compounds namely steroids, terpenoid, and tannins. Inhibitory activity of seaweed extracts on *C. albicans *biofilm formation increased as their concentration increased. The highest inhibitory effect was recorded at fungus culture treated with seaweed concentration of 25% at 24 hours of time exposure.

**Conclusions:** Seaweed *G. verrucosa *extract contained steroids, terpenoids and tannins that were able to effectively inhibit the formation of biofilm by *C. albicans *at the concentration of 25% **after 24 hours of time exposure.

## Introduction

Smoking is a common problem in both developed and developing countries, including in Indonesia. Based on a survey by the Tobacco Atlas in 2015, Indonesia has the highest number of smokers in Asia, with 66% of men in Indonesia being active smokers ^1^. Smoking can lead to addiction owing to the nicotine contents, and harm due to the presence of toxic compounds such as carbon monoxide, ammonia and tar contents in tobacco ^1^. Substances in cigarettes can also contribute for the occurrence of oral candidiasis, infection of in the mouth cavity caused by the fungus *Candida albicans* ^2^. This fungus is part of the normal flora of the human mouth, but it can become pathogenic in certain conditions, for example, due to nicotine exposure ^3^.

The *C. albicans* that infects human tissues generally form biofilm ^3^, an extracellular matrix consisting of *C. albicans* colonies ^4^. The size of the biofilm increases when the fungus was exposed to substances in cigarette smoke because cigarette contains chemicals that can initiate the growth of and nourish *C. albicans* ^5, 6^.

Currently, fluconazole and nystatin are the most effective drugs for treating oral candidiasis. Unfortunately, these drugs could result in undesired side effects. Prolonged use of fluconazole, for example, can leads to resistance ^7^ whereas high dosages of nystatin give gastrointestinal discomfort and increase plaque formation ^8^. Therefore, plant-derived antifungals may be a viable oral treatment option for candidiasis. One of these potential plants is *Gracilaria verrucosa.* This seaweed contains several bioactive compounds, including alkaloids, flavonoids, phenolics, saponins, steroids and terpenoids ^9^. Aceh Province, Indonesia, has large *G. verrucosa* resources, although this aquatic plant has not been commonly used for medicinal purposes. Hence, the objective of the present study was to examine the ability of seaweed extract to inhibit the biofilm formation of *C. albicans* isolated from the saliva of smoker.

## Methods

### Time and site

The study was conducted in August 2017 at the Laboratory of Microbiology, Veterinary Faculty, Syiah Kuala University. The *G. verrucosa* seaweeds were collected from a farmer in Pulo Aceh, Aceh Province.

### Ethics

All research protocols used in this study were approved by the Research Ethics Committee of the Dentistry Faculty of Syiah Kuala University No. 1741/UN11.1.21/TU/2017.

### Saliva collection

The saliva was collected from an active smoker who worked as administrative staff at the Faculty of Dentistry Medicine of Syiah Kuala University and voluntarily participated in this study after completing informed consent. Inclusion criterion of the volunteer was active smoker who smoked 20 cigarettes per day. Saliva was collected once by spitting into a glass jar (15 ml) right after the subject finished smoking, and added with 10 ml of PBS (0.01 M, pH 7.2). The jar was centrifuged at 10,000 rpm for 10 minutes. The precipitate was stored for the microbiological examination.

### *Candida albicans* isolation and preparation

Precipitate was cultured in ChromAgar *Candida* medium and incubated at 37°C for 2 days. The *C. albicans* fungus grew as green colonies. One colony of *C. albicans* was mixed with 5 ml of peptone in a tube and incubated at 37°C for 24 hours. Turbidity of medium was compared to a 0.5 McFarland solution standard, which was equivalent to 1.5 × 10 ^8^ CFU/ml.

### Seaweed extraction

Seaweed extraction was performed based by maceration using 96% methanol ^10^. In brief, a total of 500 g of fresh seaweeds were washed with tap water then with distilled water. Seaweed samples were at 25°C for 24 hours, chopped into small pieces (2 mm), and soaked in 1.5 liters of 96% methanol. Macerate was filtered using Whatman filter paper No. 42. Filtrate collected was concentrated using a vacuum rotary evaporator (Laborta 4003 control, Heildolph) 60°C and at a speed of 80–90 rpm for 3 days. Concentrated extract was put in a sealed dark bottle and stored at 4°C.

### Phytochemical tests

***Flavonoid test*.** A 0.5 cm magnesium plate was rinsed in 5 ml of seaweed extract, mixed with two drops of HCl, and heated by passing it over a Bunsen flame. Red or purple coloration formed on the heating indicated the presence of flavonoids ^11^.

***Alkaloid test*.** Seaweed extract (5 ml) was mixed with 8 ml of HCl and filtered. Filtrate was subjected to Mayer, Wagner and Dragendroff tests for alkaloids ^11^. This was done by mixing 2 ml of filtrate with 5 g potassium mercuric iodide (Mayer test), 2 ml of Wagner reagent, or 2 ml of bismuth potassium iodide solution (Dragendroff test). The formation of white or pale precipitates (Mayer test), brown or reddish-brown precipitates (Wagner test) and red precipitates (Dragendroff test) indicated the presence of alkaloids.

***Tannin/phenolic test*.** Two drops of 1% FeCl _3_ was added to 1 ml seaweed extract. The change in the color to a blackish green indicated the presence of tannin/phenolic ^12^.

***Saponin test*.** Seaweed extract, 1 ml, was diluted in 20 ml of distilled water and shaken vertically for 15 seconds. Persistent foaming indicated the presence of saponin content.

***Steroid test*.** Two milliliters of seaweed extract was added with 2 ml of CHCl _3_, 2 drops of H _2_S and 1 ml of CH _3_COOH. The formation of green or blue precipitates indicated the presence of steroid ^11^.

***Terpenoid test*.** Seaweed extracts (5 ml) was mixed with 2 ml of chloroform. Concentrated H _2_SO _4_, 3 ml, were carefully added. The formation of reddish brown layer at the interface of extract and chloroform solution indicated the presence of terpenoids ^13^.

### Examination of biofilm formation

Casein-peptone lecithin polysorbate broths (Merck-1117230500), 100 µl, were poured in each well of a 96-well plate, incubated at room temperature for 5 minutes and discarded by blotting the plate on paper towels 2–5 times. Fifty microliter *C. albicans* culture had turbidity was equal to 0.5 McFarland standard were added to each well and incubated for 5 minutes to attachment of fungal cells on casein. Cell mixtures were washed by aspiration. In triplicate, 50 µl of decreased concentrations of seaweed extracts (100, 75, 50, 25, 12.5 and 6.25%) were added. The same reaction using fluconazole 0.31 µg/ml was prepared as positive control. The plates were then incubated at 37°C for 24, 48 or 72 hours. Each well was added with 200 µl of 0.1% violet crystal, incubated at 25°C for 15 min, and washed three times with 200 µl of 0.01 M PBS. The crystal violet in each well was then removed by adding 100 µl of 96% ethanol for 2 min. The biofilm formation was analyzed by reading optical density of mixture using an ELISA reader at 620 nm ^14, 15^.

### Data analysis

The data obtained were subjected to Kruskal-Wallis test using SPSS software v20.0 for windows.

## Results

The results of phytochemical tests in Table 1 show that methanol extract of seaweed *G. verrucosa* contained bioactive compounds belonged to steroids, terpenoids, and tannins/polyphenols.

**Table 1.** Phytochemical contents of seaweed *Gracilaria verrucosa* extract

Substance	Reagent	Result	Indication
Alkaloid	Mayer	-	White deposit
	Wagner	-	Brown deposit
	Dragendroff	-	Red deposit
Steroid	Uji Lieberman-Burchard	+	Green or blue colors
Terpenoid	Uji Lieberman-Burchard	+	Red or purple colors
Saponin	Shuffling method	-	Stable foams
Flavonoid	0.5 Mg and HCl	-	Red or purple colors
Tannin/Phenolic	MgCl _3_	+	Dark green

**Figure 1.**
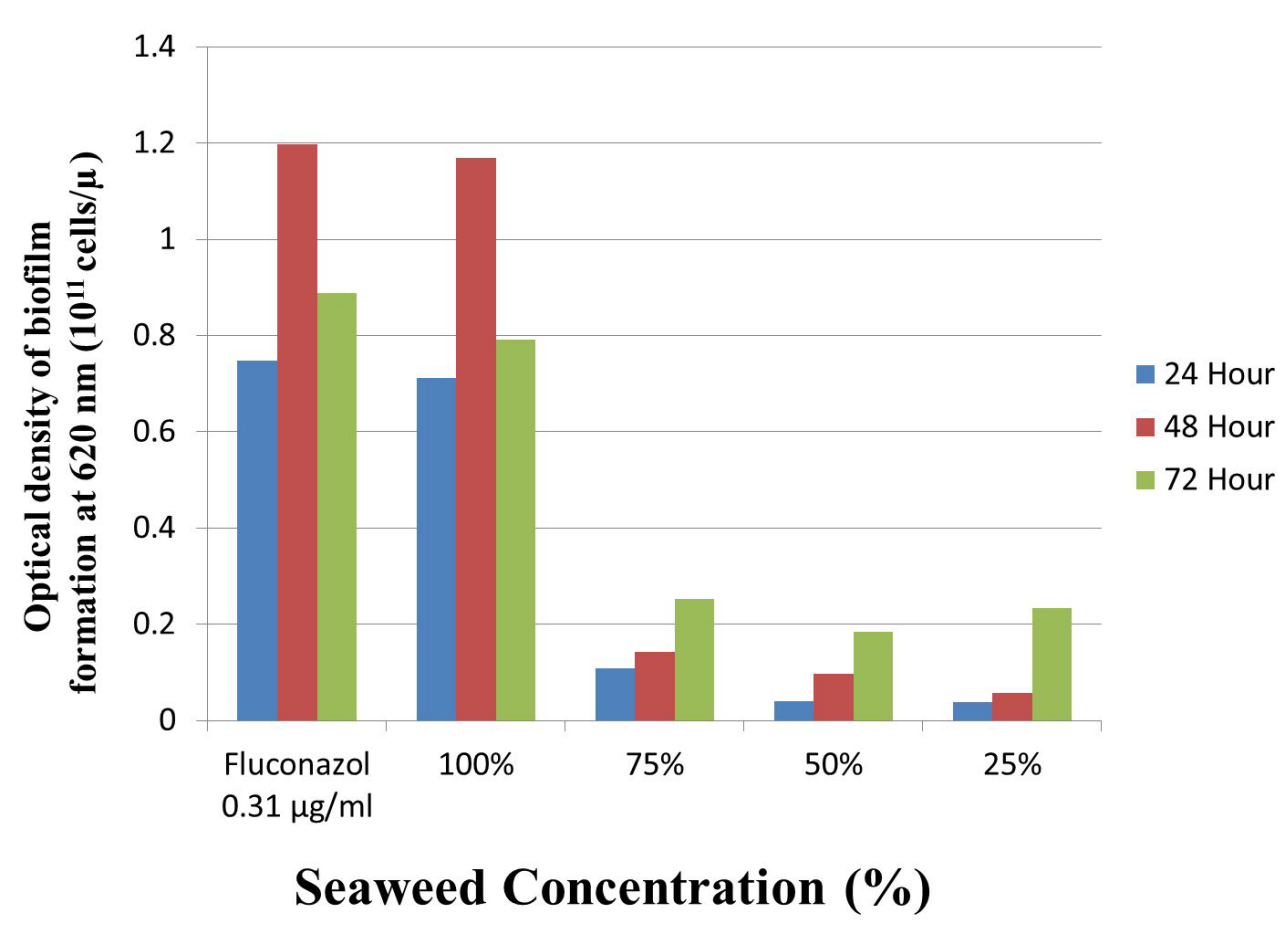
The formation of biofilm by *Candida albicans* exposed to seaweed *Gracilaria verrucosa* extract on different time exposures and concentrations.

In general, the inhibitory effect was reduced as seaweed concentration increased ( Figure 1). The highest inhibition after 24 and 48 hours time exposures was recorded from *C. albicans* culture treated with 25% seaweed extract, followed by those with 50% and 75%. The best inhibition after 75 hours time exposure, however, was found in *C. albicans* culture treated with 25% seaweed extract. In all time exposures, the inhibitory effect caused by fluconazole (control) was the worst, followed by those caused by 100% seaweed extracts, but there was no difference between these two groups. Results of Kruskal-Wallis analysis showed that seaweed extract significantly ( *p*<0.05) inhibited the formation of biofilm by *C. albicans,* indicated potential of the extract to inhibit the growth of the fungus.

The raw data of the Triplo anti-Biofilm seaweed to *C. albicans* for 24, 48 and 72 h at a wavelength 620 nm Click here for additional data file. Copyright: © 2018 Mubarak Z et al. 2018 Data associated with the article are available under the terms of the Creative Commons Zero "No rights reserved" data waiver (CC0 1.0 Public domain dedication).

## Discussion

The study showed that 25–75% seaweed extracts are promising for inhibiting the growth of *C. albicans* as shown by much lower biofilm formation after 24, 48 and 72 hours exposure compared to those caused by positive control (fluconazole) and 100% seaweed extract. This indicated potential of seaweed extract as natural anti-fungus to treat oral candidiosis caused by against *C. albicans* in smokers.

*C. albicans* is a normal micro-organism in the human mouth. This fungus, however, can be pathogenic in certain circumstances ^3^ such as in the mouth of smokers ^2^. Smoking can stimulate synthesis of HWP1, EAP1 and SAP2 proteins in *C. albicans*, causing higher virulence of the fungus. This can lead to increased formation of biofilm and finally cause oral candidiasis ^6^. Smoking also cause a decreased immune function, making individuals more susceptible to oral infections, including candidiasis ^4, 6^.

The low effectiveness of fluconazole 0.31 μg/ml, the leading choice of therapy against *C. albicans*, against this fungus was probably caused by several factors such as low therapeutic dose and resistance of the fungus to the drug. Pfaller *et al*., who complement CLSI guidelines with the EUCAST guidelines about species-specific interpretative breakpoints for fluconazole susceptibility suggested that the breakpoints for *C. albicans* are <2 mg/ml, 4 mg/ml and >8 mg ml for susceptible, susceptible dose-dependent, and resistant isolates, respectively ^16^. Dosage criteria for analyzing candida species using MIC test were susceptible (MIC < 8 μg/ml; range, 0.25 to 4 μg/ml), susceptible dose-dependent (MIC 8 – 16 μg/ml; range, >4 to <16 μg/ml), and resistant (MIC > 16 mg/ml; range, 16 to >128 mg/ml) ^17^. Fluconazole dose of 0.31 μg/ml used in this study was slightly higher than minimum dose ideally used to test antifungal resistance of the antifungal. This also meant that *C. albicans* strain isolated from smoker respondent involved in this study already developed resistance to the dose used. Increasing resistance of *C. albicans* against this fluconazole, one of the most azole antimycotics have been reported ^18, 19^. This resistance, that is assumed related to prolonged or repeated exposure to low-dose of the antifungus ^20^, must be confirmed by further study.

The stronger ability of 25–75% seaweed extracts to inhibit the formation of *C. albicans* biofilm significantly compared to those of fluconazole (positive control) was probably caused by bioactive compound presence in the extracts. These included steroids, terpenoids, and tannins/polyphenols, all of which have been known their benefit for human health. According to Sampaio *et al.* ^21^, antifungal activity of a substance strongly depends on the composition of its bioactive compounds. Steroids can kill *C. albicans* through their lypophilic properties, interfering with the formation of fungal spores and mycelium ^22^. This activity weakens *C. albicans*, inhibiting the formation of the biofilm. To function optimally, steroids require oligosaccharides that are also present in the seaweed content ^23^

Terpenoids are derivatives of saponins that may act as an antifungals by damaging organelles of the fungus and by inhibiting secretion of enzymes, leading to growth the inhibition *C. albicans* cells. Terpenoids can also damage the morphology of *C. albicans* ^24^. Tannins may inhibit chitin synthesis in *C. albicans* cell walls, leading to lost of membrane cell protection and disrupted cellular metabolism. Tannins also can inhibit ergosteron activity of *C. albicans* ^25^.

The effectiveness of seaweed extracts in inhibiting fungal growth is influenced by at least three factors, namely concentration, exposure time, and contact surface media ^26^. The present study showed that diluted concentration (25–75%) of extracts showed better inhibitory effect on the growth of *C. albicans* than the more concentrated 100% extract. Concentrated extract usually has lesser effectiveness *in vitro* due to solubility and import problems. In more aqueous condition plant extracts generally show better medicinal properties with increased concentration as shown by a study testing antifungal activity of ethanol extracts of *Syzygium jombolanum*, *Cassia siamea*, *Ordina wodier*, *Momodica charantia* and *Melia azedarach* as well as two algal species *Sargassum wightii* and *Saulerpa scalpellformis* against 25 *C. albicans* isolates *in vitro* ^27^.

This study also showed the best inhibitory effect of seaweed extracts was recorded at 24 hour of exposure. This is probably because the farnesol, a quorum-sensing molecule that has the potency to inhibit *C. albicans* growth ^28^, works effectively after 48–72 hour of exposure. 

This study, however, was still preliminary due to limited number of subject and unavailability of non-smoker, control. Virulence factor of *C. albicans* analyzed was also only biofilm formation. Since more virulence factors are possibly synthesized by the fungus under certain environmental condition, further studies are need to be conducted to investigate effect of individual bioactive compound contained in the seaweeds on the formation of biofilm and on the expression other virulence factors of *C. albicans* isolated form both smoker and non-smoker individuals.

## Conclusion

Extract of *Gracilaria verrucosa* seaweed could inhibit the growth of *C. albicans* isolated from the saliva of a smoker. The inhibitory effect decrease with the increase of concentration, and reached the highest at concentration of 25% and time exposure of 24 hours.

## Data availability

The data referenced by this article are under copyright with the following copyright statement: Copyright: © 2018 Mubarak Z et al.

Data associated with the article are available under the terms of the Creative Commons Zero "No rights reserved" data waiver (CC0 1.0 Public domain dedication). 

Dataset 1. The raw data of the Triplo anti-Biofilm seaweed to *C. albicans* for 24, 48 and 72 h at a wavelength 620 nm. DOI: 10.5256/f1000research.14879.d204270 ^29^.
