# Supplementary material for: Preliminary study on the inhibitory effect of seaweed Gracilaria verrucosa extract on biofilm formation of Candida albicans cultured from the saliva of a smoker
Source: F1000Res. 2018 Sep 18;7:684. Originally published 2018 May 31. [Version 3] doi: 10.12688/f1000research.14879.3 (PMC6107980; doi:10.12688/f1000research.14879.3)
Supplement: The raw data of the Triplo anti-Biofilm seaweed to C. albicans for 24, 48 and 72 h at a wavelength 620 nm [file f1000research-7-17646-s0000.tgz › d31bc176-ad61-44bb-94e9-e9bfe1e00734_Raw_Data.docx]

| Triplo anti-Biofilm seaweed to *Candida albicans* (24 Hours) wavelength 620 nm | | | | | | |
| --- | --- | --- | --- | --- | --- | --- |
|  |  |  |  |  |  |  |
| Dosage | 1 | 2 | 3 | Average | SDEV |  |
| 100 | 0.796 | 0.724 | 0.612 | 0.711 | 0.075707 |  |
| 75 | 0.088 | 0.13 | 0.108 | 0.109 | 0.017153 |  |
| 50 | 0.039 | 0.032 | 0.048 | 0.040 | 0.006549 |  |
| 25 | 0.034 | 0.056 | 0.027 | 0.039 | 0.012356 |  |
| 12.5 | 0.037 | 0.055 | 0.05 | 0.047 | 0.007587 |  |
| 6.25 | 0.016 | 0.064 | 0.036 | 0.039 | 0.019686 |  |
| Flukonazol | 0.53 | 0.88 | 0.832 | 0.747 | 0.154922 |  |

| Triplo anti-Biofilm seaweed to *Candida albicans* (48 Hours) wavelength 620 nm | | | | | |
| --- | --- | --- | --- | --- | --- |
|  |  |  |  |  |  |
| Dosage | 1 | 2 | 3 | Average | SDEV |
| 100 | 1.286 | 1.430 | 0.792 | 1.169 | 0.335 |
| 75 | 0.112 | 0.157 | 0.156 | 0.142 | 0.026 |
| 50 | 0.09 | 0.123 | 0.074 | 0.096 | 0.025 |
| 25 | 0.052 | 0.054 | 0.064 | 0.057 | 0.006 |
| 12.5 | 0.244 | 0.099 | 0.086 | 0.143 | 0.088 |
| 6.25 | 0.074 | 0.041 | 0.03 | 0.048 | 0.023 |
| Flukonazol | 0.87 | 0.83 | 1.891 | 1.197 | 0.601 |

| Triplo anti-Biofilm seaweed to candida albicans (72Hours) wavelength 620 nm | | | | | |
| --- | --- | --- | --- | --- | --- |
|  |  |  |  |  |  |
| Dosage | 1 | 2 | 3 | Average | SDEV |
| 100 | 0.566 | 1.440 | 0.370 | 0.792 | 0.5697 |
| 75 | 0.210 | 0.290 | 0.260 | 0.253 | 0.0404 |
| 50 | 0.150 | 0.262 | 0.142 | 0.185 | 0.0671 |
| 25 | 0.252 | 0.367 | 0.083 | 0.234 | 0.1429 |
| 12.5 | 0.101 | 0.093 | 0.076 | 0.090 | 0.0128 |
| 6.25 | 0.108 | 0.096 | 0.084 | 0.096 | 0.0120 |
| Flukonazol | 0.697 | 1.5 | 0.47 | 0.889 | 0.5412 |
