# Supplementary material for: Preliminary study on the inhibitory effect of seaweed Gracilaria verrucosa extract on biofilm formation of Candida albicans cultured from the saliva of a smoker
Source: F1000Res. 2018 Sep 18;7:684. Originally published 2018 May 31. [Version 3] doi: 10.12688/f1000research.14879.3 (PMC6107980; doi:10.12688/f1000research.14879.3)
Supplement: Peer review history

# Peer review: Referee response for version 3

**DOI:** 10.5256/f1000research.17646.r38461

**Published:** 2018-09-26

**Authors:** Cristiane Y. Koga-Ito

**Affiliations:** 1 Oral Biopathology Graduate Program, Institute of Science and Technology, São Paulo State University - UNESP, São José dos Campos, Brazil

The revised manuscript has improved. I recommend indexing of the revised manuscript as a Research Note.

I have read this submission. I believe that I have an appropriate level of expertise to confirm that it is of an acceptable scientific standard.

# Peer review: Referee response for version 3

**DOI:** 10.5256/f1000research.17646.r38462

**Published:** 2018-09-21

**Authors:** Heni Susilowati

**Affiliations:** 1 Department of Oral Biology, Faculty of Dentistry, Gadjah Mada University, Yogyakarta, Indonesia

I have read the revised version and can accept the revisions that have been made by the author insofar as this research is still an initial research.

I have read this submission. I believe that I have an appropriate level of expertise to confirm that it is of an acceptable scientific standard.

# Peer review: Referee response for version 2

**DOI:** 10.5256/f1000research.17357.r37284

**Published:** 2018-08-23

**Authors:** Shahida Mohd Said

**Affiliations:** 1 Periodontology Unit, Centre for Restorative Dentistry, Faculty of Dentistry, Universiti Kebangsaan Malaysia, Kuala Lumpur, Malaysia

Improvements made are acknowledged and approved.

I have read this submission. I believe that I have an appropriate level of expertise to confirm that it is of an acceptable scientific standard.

# Peer review: Referee response for version 2

**DOI:** 10.5256/f1000research.17357.r37282

**Published:** 2018-08-20

**Authors:** Heni Susilowati

**Affiliations:** 1 Department of Oral Biology, Faculty of Dentistry, Gadjah Mada University, Yogyakarta, Indonesia

I really appreciate the efforts of the authors who have revised the report, but I would like to add suggestions to the authors to explain why the optical density value of the treatment group treated with Fluconazole and extracts of 100% was even greater than the extracts with smaller concentrations.

I have read this submission. I believe that I have an appropriate level of expertise to confirm that it is of an acceptable scientific standard, however I have significant reservations, as outlined above.

# Peer review: Referee response for version 1

**DOI:** 10.5256/f1000research.16195.r34560

**Published:** 2018-06-27

**Authors:** Cristiane Y. Koga-Ito

**Affiliations:** 1 Oral Biopathology Graduate Program, Institute of Science and Technology, São Paulo State University - UNESP, São José dos Campos, Brazil

The manuscript by Mubarak et al. aimed to evaluate the effect of G. verrucosa extract on Candida albicans biofilm formation. The rationale of the study should be clearer. Also, there is lack of essential information throughout the text. The English should be revised.

**Specific comments**

**Introduction**

 The period “Smoking is a common problem in most developing countries, including Indonesia.”. A reference should be added. Also, smoking is not a problem only or mainly in developing countries. Please, consider revising.

 The authors stated that “This fungus is part of the normal flora of the human mouth, but it can become pathogenic in certain conditions, for example, due to nicotine exposure.”. The exposure to nicotine has been correlated with increasing in C. albicans virulence factor expression. The predisposing conditions for candidiasis are much more related to immunologic state of the host and imbalance in microflora. Please, consider revising.

 Revise the period “Infection with C. albicans will increase the formation of a biofilm of the fungus”, it is confusing.

 Revise the period “The biofilm is an extracellular matrix consisting of C. albicans colonies”, it is confusing.

 The authors stated that “high dosages of nystatin give gastrointestinal discomfort and increase plaque formation”. What do authors mean by “increase plaque formation”?

 The rationale of the study is not clear. Why did the authors select G. verrucosa? Is this plant commonly used? Why did the authors decide to use saliva from a smoker individual?

**Methods**

 Revise the period “C. albicans was extracted from the saliva”, C. albicans was isolated from saliva.

 The authors reported that “G. verrucosa seaweed was collected from a farmer in Pulo Aceh, Aceh Province.”. More information on the plant, the exact location it was collected from, the period of the year, identification procedure (how and who did the identification?), registration in herbarium, number of voucher should be included in the text.

 Please, revise the period “Saliva was collected by spitting into a glass jar (15 ml), then 1 ml PBS (0.01 M, pH 7.2) was added to the jar.” Why did 1 ml of PBS added to the saliva? What was the final volume of saliva collected? Was saliva stimulated?

 The authors stated that “If the colour of a colony was green, this indicated that the colony was C. albicans.”. However, the color of the colony in CHROMagar is only a presumptive test and phenotypic or genotypic definitive identification should be done.

 The inclusion of a reference strain is highly needed.

 Include the number of experiments/replicates performed.

 The inclusion of more clinical isolates from non-smokers patients is needed.

 The methodology of activity of extract on biofilm formation is not clear. Why and how peptone was removed from the wells? Why did the fungal suspension left in the wells for 5 min? How 100% extract concentration was obtained in the well (you already has the broth inside the well)? Why the concentration of 0.31 was chosen for fluconazole?

 Figure 1 should be revised. Note that optical density at 620 nm is higher after treatment with 100% extract when compared to the other concentrations.

 Discussion and Conclusion sections should be revised after the revision of the aforementioned points.

I have read this submission. I believe that I have an appropriate level of expertise to confirm that it is of an acceptable scientific standard, however I have significant reservations, as outlined above.

# Peer review: Referee response for version 1

**DOI:** 10.5256/f1000research.16195.r34561

**Published:** 2018-06-19

**Authors:** Shahida Mohd Said

**Affiliations:** 1 Periodontology Unit, Centre for Restorative Dentistry, Faculty of Dentistry, Universiti Kebangsaan Malaysia, Kuala Lumpur, Malaysia

Overall fair manuscript with sound finding and relevant area of study. Can much be improved with grammar check and essential scientific writing reorganisation especially in Introduction and Discussion section. Inclusion of results for negative control (untreated biofilm) would critically improve Results presentation and appreciation of findings. Lacks relevant information on findings between smoker and non-smoker in the study that may not strongly support the conclusion on the anti-fungal effect of agent on smoker.

I have read this submission. I believe that I have an appropriate level of expertise to confirm that it is of an acceptable scientific standard, however I have significant reservations, as outlined above.

# Response

**Published:** 2018-08-05

**Authors:** Zaki Mubarak

**Affiliations:** Syiah Kuala University, Indonesia

Some improvement in grammar, sentences and writing reorganization have been done as a response to reviewer Dr Shahida M. Said. Some details in methods and analysis have also been provided to make better readibility and potential reproducibility of the work. As a result the work in this study is much better presented. These improvements can be found in track changes.

As this study is still preliminary comparison between smoker and non-smoker has not been performed yet, but the finding clearly show potential of use of seaweed extract to treat oral candidiasis.

# Peer review: Referee response for version 1

**DOI:** 10.5256/f1000research.16195.r34557

**Published:** 2018-06-12

**Authors:** Heni Susilowati

**Affiliations:** 1 Department of Oral Biology, Faculty of Dentistry, Gadjah Mada University, Yogyakarta, Indonesia

This preliminary research is interesting enough to be developed but there are some things that need to be reconsidered:

1. ABSTRACT

- Research background that written on the abstract (lines 6 and 7) does not match the purpose of the study. The sentence can be interpreted in contrast to the antifungal potential possessed by Garcinia verrrucosa. It is dubious to investigate the potency of biofilm inhbition effect if Candida albicans more tolerant to Gracilaria extract.  

        2. METHODS

 Methods need to explain the following:

- Systemic conditions and state of teeth and oral soft tissue volunteers,
- The predestined determination of the Gracilaria verrucosa plant should be mentioned.
- Were the culture washed after the incubation period on treatment?
-  How many times an experiment produces a representative result?

        3. RESULTS

- Interpretation of the results is confusing; as far as I know the higher the optical density value the more biofilms are formed. The results in Fig. 1 show that 100% and Fluconazole extracts have higher optical densities rather than the lower concentration of extracts, as far as I know this shows that the mass of biofilms formed in the group is higher. Please observe the methods and results of research reported by Sebaa et al 2016.
- The statistical method used was only Kruskal-Wallis, is there a multiple difference analysis? Researchers need to discuss the effect of antibiofilm extract at lower concentrations, because of course 100% extract is not a good recommendation for subsequent experimental use.

I have read this submission. I believe that I have an appropriate level of expertise to confirm that it is of an acceptable scientific standard, however I have significant reservations, as outlined above.

## References

[1] SebaaSHizetteNBoucherit-OtmaniZCourtoisP: Dose‑dependent effect of lysozyme upon Candida albicans biofilm. *Mol Med Rep*.2017;15(3) : 10.3892/mmr.2017.6148 1135-1142 10.3892/mmr.2017.6148 28138698PMC5367326

# Response

**Published:** 2018-08-05

**Authors:** Zaki Mubarak

**Affiliations:** Syiah Kuala University, Indonesia

**Abstract**

Comments from reviewer :

The objective has been clarified

 Action done:

We think aims of study still need to be stated

Rearrangement and sentences improvement were done in some sections of abstract

**Introduction**

Comments from reviewer :

No comments

Action done:

Grammar check, revise and improvement were done to provide more concise and clear sentences/statements

**Material and method**

Comments from reviewer :

Need clarification on sentence:

“This volunteer has a bad OHIS4” (time and place)

“.Then microtiter wells plate wells were washed three times with 200µl of PBS buffer and dried for 15 minutes.”(biofilm examination)

“This examination was repeted three times with triplo method”( biofilm examination)

Action done:

Sentences improvement, grammar check and correction had been done throughout this section to provide better readability, clarity and possible reproducibility

**Results**

Comments from reviewer :

No comments

Action done:

Rewritten this part due to misinterpretations we did in the first previous manuscript about which treatment gave best inhibition effect on *C. albicans*

**Discussion**

Comments from reviewer :

No specific comments

Action done:

Rewritten has been done to this part in realtion to due to changes in results presentation

Sentences improvement, grammar check and reorganization had been done throughout this section to provide better readability and clarity

**Conclusion**

Comments from reviewer :

No specific comments

Action done:

This part had been rewritten in relation to the revision performed in result and discussion.
